# Influence of COVID-19 pandemic on pregnancy and fertility preferences among the residents of the United Arab Emirates (UAE)

**DOI:** 10.7189/jogh.14.05002

**Published:** 2024-02-09

**Authors:** Subhashini Ganesan, Latifa Mohammed Al Ketbi, Flavia Martinez Cantarutti, Nawal Al Kaabi, Mohammed Al Mansoori, Mariam Rashed Al Saedi, Fatima Ibrahim Al Blooshi, Ruqaya Abdulla Al Nuaimi, Marie Ibrahim, Islam Eltantawy, Fahed Al Marzooqi, Walid Abbas Zaher

**Affiliations:** 1 G42 Healthcare, Abu Dhabi, United Arab Emirates; 2 Insights Research Organization and Solutions (IROS), Abu Dhabi, United Arab Emirates; 3 Ambulatory Healthcare Services, SEHA, Abu Dhabi, United Arab Emirates; 4 United Arab Emirates University, Abu Dhabi, United Arab Emirates; 5 Khalifa University, Abu Dhabi, United Arab Emirates

## Abstract

**Background:**

The coronavirus disease 2019 (COVID-19) significantly impacted the lifestyles of millions of people, with new challenges arising as the pandemic progresses. However, little attention has been given to issues like fertility intentions and pregnancy planning during COVID-19. Consequently, we aimed to investigate the influence of the pandemic on pregnancy and fertility decisions among the residents of the United Arab Emirates (UAE).

**Methods:**

We surveyed UAE residents of reproductive age between November 2021 to June 2022 via the Google Forms platform and collected data on demographics, associated health conditions, COVID-19 infections, as well as plans for pregnancy and fertility intentions. We presented data through descriptive statistics (frequencies and percentages) and used Pearson’s χ^2^ test to compare the characteristics of participants who reported that the COVID-19 pandemic has influenced their fertility preferences with those who reported that it had not.

**Results:**

Overall, 564 participants completed the survey, of whom 115 (20.4%) stated that the COVID-19 pandemic had influenced their fertility preferences. Meanwhile, 234 (41.5%) reported previous history of COVID-19 infection; regarding post-COVID-19 infection symptoms, 53 (22.6%) reported decreased libido and 40 (17%) reported trouble in conceiving a baby. Participants who were ≤30 years of age were less likely to be influenced by the COVID-19 pandemic on their decision on fertility compared to those >30 years of age. Factors like education, income, chronic health conditions, and previous history of COVID-19 infection or vaccination did not have any significant effect on the COVID-19 pandemic influence on fertility preferences.

**Conclusions:**

The COVID-19 pandemic has brought in new challenges which could affect fertility and this needs to be studied further for planning effective measures.

The coronavirus disease 2019 (COVID-19) pandemic, caused by the novel severe acute respiratory coronavirus 2 (SARS-CoV-2), impacted people’s personal and social lives globally to an as-of-yet unknown extent [1], including their lifestyles, social mandates, economic situation, and perception of health and wellbeing. These changes have also affected people’s fertility preferences and willingness to have children. Reports have shown how the pandemic has influenced the sexual and reproductive health among women [2]. COVID-19 infection can affect anyone, regardless of age and gender, but the susceptibility increases with people of compromised immune status and pre-existing comorbidities. Thus, COVID-19 infections seem to result in more adverse outcomes in pregnant than in non-pregnant women [3]. Studies have also found severe morbidity in relation to SARS-CoV-2 infection in pregnancy, as well as adverse pregnancy outcomes such as pregnancy loss, preterm delivery, and laboratory evidence of vertical transmission [4–7].

Researchers have hypothesised that the increased risk for complications from SARS-CoV-2 infection in pregnant women is related to the higher angiotensin-converting enzyme 2 (ACE2) expression, leading to vasoconstriction, inflammation, and pro-coagulopathic effect [8]. The SARS-CoV-2 virus can affect these ACE2 receptors, which are also present in the testes, and can thus possibly lead to severely damaged male sexual development for younger males and infertility in adult males [9,10]. However, there is yet no evidence of SARS-CoV-2 affecting reproductive organs or fertility in females.

These effects of COVID-19 infection on pregnancy, along with the social and economic changes during the pandemic, have significantly impacted people’s fertility preferences. Couples have expressed their decisions to plan pregnancy later or wanted fewer children because of the pandemic. More than 40% women have noted a change in plans about when to have children, and about one third wanted to get pregnant at a later time [2,11]. These decisions have also been influenced by a fear of COVID-19 vaccines, as women, particularly pregnant ones or those planning to conceive in the next six months, were found to be hesitant to accept the currently available mRNA COVID-19 vaccines [12]. However, there is no evidence that the COVID-19 vaccination affects pregnancy or harms the foetus or that it can impact fertility [13-15].

There are also trials ongoing to investigate the effects of COVID-19 vaccination on semen parameters [16], yet available evidence does not suggest that COVID-19 vaccination affects pregnancy or fertility. Even so, the anxiety, fear of vaccination, and the wide-reaching misinformation available on social media seem to have impacted individuals’ decisions on pregnancy and fertility preferences. Thus, several factors related to the COVID-19 pandemic have likely influenced people’s intention on pregnancy and fertility preferences, leading those who intended to have children before the pandemic to reconsider their decisions [17]. Through this survey, we sought to understand the pandemic’s impact on fertility preferences among people of reproductive age in the United Arab Emirates (UAE).

## METHODS

### Study design and setting

We conducted an online survey-based cross-sectional study between November 2021 to June 2022 among residents of the UAE to identify fertility preferences and the influence of the COVID-19 pandemic on pregnancy and fertility-related decisions.

The questionnaire was designed to elicit responses on the participants’ perception of the influence of COVID-19 on fertility preferences, history of COVID-19 infection, vaccination, and changes following COVID-19 infection. It was developed and content validated among experts from various fields, including family medicine, infectious disease, obstetrics, and public health. The validated questionnaire was then piloted among the interns at the family medicine department to assess for clarity, relevance, ambiguity, feasibility, readability, consistency of style, and formatting. The first section collected details on demographical characteristics, including age, gender, education, occupation, and income. The second part explored chronic health conditions, history of COVID-19 infection, vaccination, changes following COVID-19 infection, and complications. The questionnaire was distributed using Google forms.

We selected the study participants through convenience sampling among people attending the family health clinics. We then use the snowballing method and asked participants to share the survey among their friends and family in the UAE who were of reproductive age. The forms were limited to a single response to avoid duplication. We screened the submitted forms for completion and sent only the completed ones for analysis. We obtained informed consent electronically during the online survey and included only the individuals who agreed to participate.

### Study participants

UAE residents of reproductive age (21**–**45 years), regardless of nationality or gender, were included in the survey; 773 individuals responded and submitted their forms. However, we considered only 545 participants who had fully completed the questionnaire and answered the questions on the influence of the COVID-19 pandemic on fertility preferences for further analysis.

### Statistical analysis

We did not calculate a definitive sample size, as we aimed to collect as much data as possible over the study period. We presented all data descriptively in frequencies and percentages, analysing them using Pearson’s χ^2^ to compare the characteristics of participants who reported that the COVID-19 pandemic has influenced their fertility preferences and those who reported that it had not. We used the results of the Pearson’s χ^2^ test and the related *P*-values to assess the effect of different factors on the outcome of the study, assuming a 5% significance level. We also calculated odds ratios (ORs) with 95% confidence intervals (CIs). We conducted all analyses in Stata, version 28.0 (StataCorp LLC, College Station, TX, USA).

## RESULTS

### Descriptive characteristics

#### Participants details

A total of 564 participants completed the study, of whom 486 (86.2%) were female. The low response rate of males (13.8%) was a major reason for this skewed sex distribution. The mean age of the participants was 32.1 years (standard deviation = 7.5). About 70% were graduates and about 50% of them had a monthly income of ≥10 000 AED. Overall, 120 (21.3%) of the participants had one or more chronic health conditions (**Table 1**).

**Table 1:** Demographical variables of the study participants (n = 564)

	n (%)
**Age group**	
≤30 y	248 (44.0)
>30 y	316 (56.0)
**Gender**	
Male	78 (13.8)
Female	486 (86.2)
**Educational qualification**	
High school and below	168 (29.8)
Bachelor/postgraduate	396 (70.2)
**Income as AED per month**	
<10 000	275 (48.8)
≥10 000	289 (51.2)
**History of COVID-19 infection**	
Yes	234 (41.5)
No	330 (58.5)
**Chronic health conditions**	
Yes	120 (21.3)
No	444 (78.7)
**COVID-19 vaccination status**	
Vaccinated	526 (93.3)
Not vaccinated	38 (6.7)

y – years

#### Fertility preferences, COVID-19 infections, and vaccination

Out of 564 individuals, 115 (20.4%) stated that the COVID-19 pandemic had influenced their decision on fertility preferences; 19 (3.4%) were pregnant during the survey, 178 (31.6%) were planning to conceive, and 367 (65.1%) did not want to conceive during the pandemic. In total, 234 (41.5%) participants reported previous history of COVID-19 infection; 53 (22.6%) reported decreased libido and 40 (17.0%) reported trouble in conceiving a baby.

Among the study participants, 526 (93.3%) were vaccinated against COVID-19. Among the remaining 38 (6.7%), 8 (21.1%) said they feared the vaccine could affect pregnancy or the foetus and 5 (13.2%) believed that the vaccine can cause infertility and affect childbearing abilities. The other individuals mentioned reasons such as disbelief in vaccines, other side effects of vaccines, that they did not feel it necessary to receive vaccine, and other reasons.

### Effect of demographic factors, COVID-19 infection, and vaccination on the changes in fertility preferences due to COVID-19 pandemic

Among factors like educational status, income, chronic health conditions, vaccination status, and previous history of COVID-19 infection, only age had a significant effect on the influence of the COVID-19 pandemic on fertility preferences. Participants who were ≤30 years old were less likely to be influenced by the COVID-19 pandemic on their decision on fertility compared to participants who were more than 30 years of age (OR = 0.60; 95% CI = 0.38–0.90, *P*-value <0.05) (**Table 2**).

**Table 2:** Association of factors on influence of the COVID-19 pandemic on fertility preferences (n = 564)

	Impact of COVID-19 pandemic influence on fertility preferences		OR (95% CI)	*P-*value
	**Yes**	**No**		
**Education**				
High school and below	32 (19.0)	136 (81.0)	0.89 (0.56–1.39)	0.649
Bachelor/postgraduate	83 (21.0)	313 (79.0)		
**Income as AED per month**				
<10 000	48 (17.5)	227 (82.5)	0.70 (0.46–1.06)	0.095
≥10 000	67 (23.2)	222 (76.8)		
**Age group**				
≤30 y	39 (15.7)	209 (84.3)	0.60 (0.38–0.90)	0.016
>30 y	76 (24.1)	240 (75.9)		
**Vaccination status**				
Vaccinated	106 (20.2)	420 (79.8)	0.81 (0.38–1.77)	0.676
Not vaccinated	9 (23.7)	29 (76.3)		
**Previous COVID-19 infection**				
Yes	51 (21.8)	183 (78.2)	1.16 (0.77–1.75)	0.525
No	64 (19.4)	266 (80.6)		
Chronic health conditions				
**Yes**	28 (23.3)	92 (76.7)	1.25 (0.77–2.03)	0.373
**No**	87 (19.6)	357 (80.4)		

CI – confidence interval, OR – odds ratio, y – years

## DISCUSSION

In this study, we assessed the impact of the COVID-19 pandemic on fertility preferences among UAE residents. Throughout the COVID-19 pandemic, people had many doubts and fears regarding the effect of the virus on their health, especially on the impact on fertility, pregnant women, and foetuses. Twenty per cent of our participants stated that the COVID-19 had influenced their decision on fertility preferences, which is comparable to a survey in the USA, where approximately one third (29.6%) of the participants changed their fertility preferences because of the COVID-19 pandemic [18]. However, in another study conducted in the USA, more than 40% of the participants reported having changed their plans about having children due to the COVID-19 pandemic [2]. This difference could be due to the different study periods; while we conducted our study during the last quarter of 2021 and mid-2022, the US study was conducted much earlier in the pandemic, in early 2020. Likewise, the impact of the pandemic on the economy may differ between countries, and individuals’ pregnancy plans may rely on such factors.

About 65% of our study participants did not want to conceive because of the pandemic. A similar study conducted in Italy showed 37.3% of the people who were planning to have a child before the pandemic changed their intention to do following its outbreak. The main reasons were the consequences of COVID-19 on pregnancy and foreseen economic difficulties [17].

Our results showed that the fertility preferences and pregnancy plans of individuals ≥30 years of age were more influenced by the pandemic than those of their younger counterparts aged <30 years. These results were similar to those of a survey done in the USA, which showed that participants who changed their fertility preferences because of the COVID-19 pandemic were older compared to those who did not [18].

In this study, factors like gender, education, income, associated chronic health conditions, previous COVID-19 infections, or vaccination status did not have any significant effect on COVID-19 pandemic on fertility preference. However, other studies have shown that women with a lower income were more likely to report changes in fertility preferences during the pandemic than those with a high income [2]. In the aforementioned study conducted in Italy, females predominantly changed their desire for child during the pandemic compared to males [17]. The reasons we could not establish such gender differences could be the low proportion of men in our sample.

In our study, about 20.0% of the COVID-19 survivors reported decreased libido and difficulty in conceiving a baby following COVID-19 infection. Studies have supported similar sexual and reproductive health issues in COVID-19 survivors [19]. Meanwhile, studies among men showed that 65.0% of COVID-19 patients have reported a loss of libido and abnormal sex hormone secretion [20,21]. Likewise, the pandemic as such has had an impact on the people’s sexuality and led to increased rates of sexual dysfunction [19,22].

Most of our study participants (93%) were vaccinated against COVID-19; participants who were not vaccinated expressed fear of the vaccine affecting their fertility and or their foetus. COVID-19 vaccinations have been found to be safe in pregnancy are highly recommended for pregnant women, as COVID-19 infection during pregnancy can cause serious consequences both in the mother and the foetus [23–25]. These fears could have also played a role in fertility preferences during the pandemic, which is beyond the scope of our research.

## CONCLUSIONS

The COVID-19 pandemic has impacted the fertility preferences of 20% of the population, especially those ≥30 years of age. There are reports of sexual issues among the COVID-19 survivors and the fear of impact of vaccines on reproductive health; however, the influence on these factors on fertility preferences during pandemic requires further exploration. The COVID-19 pandemic has brought in new challenges which could affect fertility and this needs to be studied further for planning effective measures.
